# Spectrum of findings on magnetic resonance imaging of the brain in patients with neurological manifestations of dengue fever

**DOI:** 10.1590/0100-3984.2016.0048

**Published:** 2017

**Authors:** Tejeshwar Singh Jugpal, Rashmi Dixit, Anju Garg, Swati Gupta, Virendra Jain, Ronak Patel, Shobhit Agarwal

**Affiliations:** 1 MD, Department of Diagnostic Radiology, Maulana Azad Medical College, New Delhi, India.; 2 MD, Department of General Medicine, Maulana Azad Medical College, New Delhi, India.

**Keywords:** Dengue, Encephalitis, Magnetic resonance imaging, Leukoencephalitis, acute hemorrhagic, Cerebellar diseases/diagnosis, Dengue, Encefalite, Ressonância magnética, Leucoencefalite hemorrágica aguda, Doenças cerebelares/diagnóstico

## Abstract

**Objective:**

To describe the spectrum of magnetic resonance imaging (MRI) findings in patients with neurological manifestations of dengue.

**Materials and Methods:**

We included nine patients with dengue fever (three females and six males; age range, 9–30 years), all of whom presented with neurological manifestations. The MRI examinations, performed in 1.5 T or 3 T scanners, included T1-weighted, T2-weighted, and fluid-attenuated inversion recovery (FLAIR) sequences. Diffusion-weighted imaging with apparent diffusion coefficient mapping was also employed. Fast low-angle shot and susceptibility-weighted gradient-recalled echo sequences, as well as contrast-enhanced T1-weighted scans, were also obtained in order to assess parenchymal enhancement. MRI scans were analyzed for lesion distribution and imaging features.

**Results:**

All patients showed areas of altered signal intensity that appeared as hyperintensity on T2-weighted and FLAIR sequences. The most commonly affected site was the basal ganglia-thalamus complex. Other affected sites were the cerebellum, cerebral cortex, white matter, and brainstem. In all cases, we observed patchy areas of restricted diffusion and focal areas of hemorrhage.

**Conclusion:**

Dengue encephalitis commonly affects the basal ganglia, thalamus, cerebellum, cerebral cortex, and white matter. Therefore, MRI should be an indispensable part of the evaluation of patients with neurological complications of dengue fever.

## Abstract (pt)

**Objetivo:**

Descrever o espectro dos achados de ressonância magnética (RM) em pacientes com manifestações neurológicas de dengue.

**Materiais e Métodos:**

Foram incluídos nove pacientes com dengue (três do sexo feminino e seis do sexo masculino; faixa etária: 9–30 anos), todos com manifestações neurológicas. Os exames de RM, realizados em aparelhos de 1,5 T ou 3 T, incluíram sequências ponderadas em T1 e em T2, assim como *fluid-attenuated inversion recovery* (FLAIR). Também foi empregada a imagem ponderada em difusão com mapeamento de coeficientes de difusão aparente. Além disso, foram obtidas sequências gradiente-eco ponderadas por suscetibilidade e em *fast low-angle shot*, bem como imagens ponderadas em T1 pós-contraste, para avaliar o realce parenquimatoso. As imagens de RM foram analisadas quanto à distribuição de lesões e características de imagens.

**Resultados:**

Todos os pacientes apresentaram áreas de intensidade de sinal alteradas que apareceram como hiperintensidade em sequências ponderadas em T2 e sequências FLAIR. O local mais comumente afetado foi o complexo gânglios basais-tálamo. Outros locais afetados foram o cerebelo, o córtex cerebral, a substância branca e o tronco encefálico. Em todos os casos observamos áreas irregulares de difusão restrita e áreas focais de hemorragia.

**Conclusão:**

A encefalite por dengue geralmente afeta os gânglios basais, o tálamo, o cerebelo, o córtex cerebral e a substância branca. Portanto, a RM deve ser uma parte indispensável da avaliação de pacientes com complicações neurológicas da dengue.

## INTRODUCTION

Dengue fever is an arthropod-borne febrile viral illness commonly seen in tropical and subtropical countries. It has a highly variable presentation, ranging from mild clinical symptoms to complications such as dengue shock syndrome and dengue hemorrhagic fever, which can be life-threatening. Dengue is a single-stranded virus that belongs to the genus *Flavivirus*, has four serotypes (DEN1 through DEN4), and is transmitted to humans by the *Aedes aegypti* mosquito^(1-3)^. Although the dengue virus is generally thought to be non-neurotrophic, the incidence of early neurological manifestations of dengue has increased in recent years^(4)^. The reported incidence of neurological complications of dengue has been found to vary between 0.5% and 6.2%^(3)^. The neurological complications of dengue are broadly classified into the following categories^(5,6)^: encephalopathy secondary to systemic involvement such as coagulopathy, hepatic failure, and systemic hypotension; direct neuronal invasion by the virus causing encephalitis; and immune complex-mediated vasculitis and demyelinating process such as acute disseminated encephalomyelitis (ADEM).

There have been only a few studies describing magnetic resonance imaging (MRI) findings in patients with dengue fever^(7-9)^. Here, we describe the spectrum of MRI features in patients with serologically confirmed dengue and neurological manifestations.

## MATERIALS AND METHODS

Using MRI, we evaluated nine patients (9–30 years of age) with dengue fever and neurological manifestations including seizures, altered sensorium, and ataxia. The sample comprised three females and six males. The cases were diagnosed based on clinical features, laboratory test results, and serology, all serum samples testing positive for immunoglobulin M (IgM) antibodies or for nonstructural protein 1 antigen. All of the patients gave written informed consent.

The MRI examinations were performed in dedicated 1.5 T or 3 T MRI scanners. All of the patients underwent imaging during the acute phase of the illness (between day 3 and day 6). The MRI scans included T1-weighted fast spin-echo, T2-weighted, and fluid attenuated inversion recovery (FLAIR) sequences. Diffusion weighted (DWI) images were acquired using single-shot fast spin-echo echo-planar sequences with sensitizing gradients applied in all three orthogonal planes with b factors of 500 s/mm^2^ and 1000 s/mm^2^. We also generated apparent diffusion coefficient maps using the software supplied by the vendor. To identify hemorrhagic foci, we also acquired gradient-recalled echo (GRE) fast low-angle shot sequences (in six patients) and susceptibility-weighted imaging (SWI) sequences (in three patients). Contrast-enhanced T1-weighted scans were also obtained in all cases. 

## RESULTS

All nine patients presented with high-grade fever, headache, and altered sensorium. Seizures were observed in five patients. Ataxia was seen in three patients, one of whom also had severe vertigo, tremors, and nystagmus. All the patients had normal liver and kidney function. At the time of imaging, we also determined the platelet count. The lowest platelet count noted during the study was 21,000 cells/mL. The clinical findings are summarized in Table 1.

**Table 1:** Summary of clinical findings and relevant lab investigation.

Patient No.	Age (years)	Sex	Neurological symptoms	Duration of illness (days)	Platelet count (cells per mL)	Serum NS1 antigen/IgM anti-dengue antibody
1	9	Female	Fever, headache, seizure	5	25,000	Positive
2	30	Male	Fever, headache, seizure, altered sensorium	4	20,000	Positive
3	17	Male	Fever, headache, altered sensorium,	5	40,000	Positive
4	22	Male	Fever, headache, seizure, ataxia	5	36,000	Positive
5	20	Male	Fever, headache, seizure, altered sensorium	4	30,000	Positive
6	21	Male	Fever, headache, altered sensorium, seizure	4	39,000	Positive
7	25	Male	Fever, headache, ataxia, nystagmus, vertigo, tremors	6	29,000	Positive
8	16	Female	Fever, headache, altered sensorium	3	68,000	Positive
9	22	Female	Fever, headache, ataxia, altered sensorium	5	21,000	Positive

The anatomical distribution of the lesions and their MRI characteristics are summarized in Table 2. All the patients showed abnormal signal intensities on MRI. The basal ganglia-thalamus complex was involved in seven cases: isolated involvement of the basal ganglia in one; involvement of the basal ganglia and thalamus in two; and isolated involvement of the thalamus in four. Cerebral and cerebellar involvement was also seen in four patients. The lesions appeared hyperintense on T2-weighted and FLAIR sequences. All cases showed patchy areas of restricted diffusion. The GRE/SWI sequences showed focal areas of blooming suggesting hemorrhage in all cases. On contrast-enhanced images, subtle enhancement was seen in six patients.

**Table 2:** MRI findings in nine patients with dengue encephalitis.

Patient No.	Cerebrum	Ganglio-thalamic complex	Brain stem	Cerebellum
1	-	+ (RD, Hg, E)	-	-
2	+ (RD, E)	+ (RD, Hg, E)	-	-
3	+ (RD, Hg, E)	+ (RD, Hg, E)	+ (RD, Hg, E)	-
4	-	+ (RD, Hg, E)	-	+ (RD, Hg, E)
5	+ (RD, Hg, E)	+ (RD, Hg, E)	-	-
6	+ (RD, Hg)	-	-	+ (RD, Hg)
7	-	-	-	+ (RD, Hg)
8	-	+ (RD, Hg, E)	-	-
9	+(RD)	+ (RD, Hg)	+ (RD)	+ (RD, Hg)

-, Absent; +, present; RD, restricted diffusion on DWI; Hg, hemorrhagic foci; E, enhancement on contrast-enhanced MRI.

## DISCUSSION

Dengue fever usually presents as febrile myalgia, arthralgia, frontal/retro-orbital headache, nausea, vomiting, and rash. Dengue fever is typically accompanied by thrombocytopenia. In its severe form, dengue can manifest as dengue hemorrhagic fever or dengue shock syndrome^(10)^.

The dengue virus was initially considered to be non-neurotropic virus. However, there has been an increase in the incidence of neurological manifestations in patients with dengue fever. Recent studies in which the dengue IgM antibody was isolated in cerebrospinal fluid suggest that the dengue virus is capable of direct neuroinvasion^(11)^. It has also been observed that the DEN2 and DEN3 serotypes have the greatest proclivity for producing neurological complications^(12)^. The neurological complications of dengue fever can be broadly grouped into three categories: direct viral neurotropism (encephalitis, meningitis, and myelitis); systemic complications (encephalopathy and ischemic or hemorrhagic stroke; and postinfectious complications (ADEM and myelitis). Dengue encephalitis is now recognized as a clinical entity which is a leading cause of encephalitis in endemic regions^(13-15)^.

MRI plays an important role in identifying the exact anatomical area of involvement and substantiating a diagnosis of dengue encephalitis in patients with neurological manifestations. There have been few studies describing the neuroimaging findings of dengue encephalitis on MRI scans. There have been isolated case reports suggesting that the commonly affected regions of brain include the basal ganglia, thalamus, temporal lobes, hippocampus, cerebellum, and cerebral white matter^(7,8,16-19)^. This is similar to what we encountered in our case series, in which the most common site of involvement was the basal ganglia-thalamus complex (in seven patients), followed by the cerebrum (in five) and cerebellum (in four).

**Figure 1:**
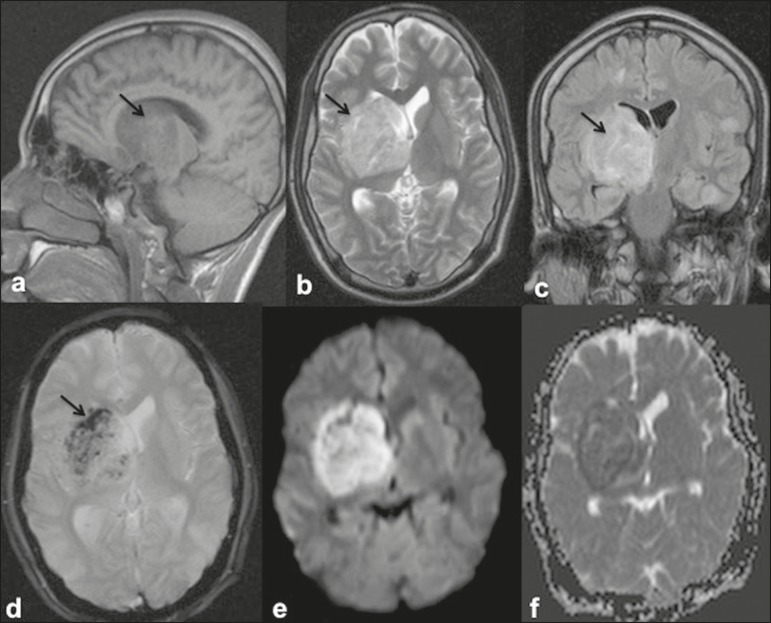
16-year-old female with dengue encephalitis. Sagittal T1-weighted image (a) showing hypointensity involving the right basal ganglia-thalamus complex (arrow), which appears hyperintense on an axial T2-weighted image (b) and a coronal FLAIR image (c), with an associated mass effect, as evidenced by effacement of the frontal horn of the right lateral ventricle. Multiple hemorrhagic foci can be seen within the lesion on a GRE fast low-angle shot sequence (d) with areas of restricted diffusion apparent on DWI (e) and apparent diffusion coefficient maps (f).

**Figure 2:**
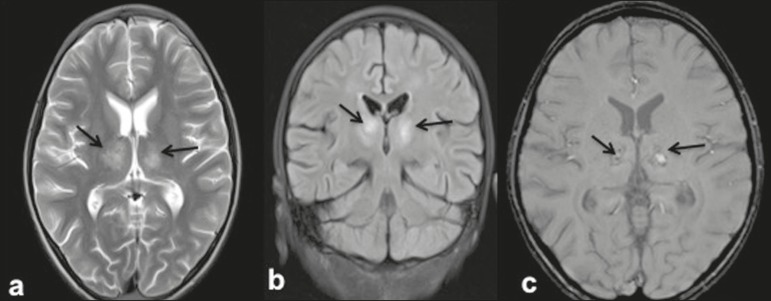
9-year-old female with dengue encephalitis. Axial T2-weighted image (a) and coronal FLAIR image (b) showing a hyperintense signal in bilateral thalami (arrows). SWI image (c) showing hemorrhagic foci in the same region (arrows).

In our case series, all of the lesions appeared hypointense on T1-weighted sequences, hyperintense on T2-weighted sequences with evidence of patchy areas of restricted diffusion. We also encountered foci of blooming on GRE/SWI sequences, which was indicative of hemorrhage within the lesions, in all patients (Figures 1 and 2). In the literature, focal lesions caused by dengue appear hyperintense on T2-weighted images and hypointense on T1-weighted images, similar to what we encountered in our studies^(7-9)^. The DWI characteristics of the lesions have been reported in only a few previous studies. In those studies, the lesions showed hyperintense signals on DWI, the diffusion being restricted in some cases and facilitated in others^(7,8)^. In our case series, all of the focal lesions showed restricted diffusion. Hemorrhagic foci within the lesions have also rarely been reported in dengue encephalitis^(7,9)^. On contrast-enhanced T1-weighted sequences, six of our nine patients showed minimal patchy heterogeneous enhancement within the lesions. This could be attributed to the fact that the permeability of the blood-brain barrier is reportedly increased in dengue infection^(15)^.

The common differential diagnoses of the MRI neuroimaging findings in patients with dengue encephalitis include Japanese encephalitis, herpes simplex encephalitis, and ADEM. The typical anatomical sites include the basal ganglia-thalamus complex (bilaterally) in Japanese encephalitis and the temporal/basifrontal lobes (also bilaterally) in herpes simplex encephalitis^(20,21)^. Hemorrhagic foci are characteristic of herpes encephalitis but are usually not encountered in Japanese encephalitis. However, hemorrhagic foci within the basal ganglia-thalamus complex have been rarely reported in cases of Japanese encephalitis^(22,23)^. Therefore it might be difficult to differentiate among these different types of viral encephalitis on the basis of MRI findings alone. Analysis of the cerebrospinal fluid and clinical profile might provide clues to the specific clinical entity. In a region where dengue is endemic, the possibility of dengue encephalitis should be borne in mind as a potential differential diagnosis, especially during dengue outbreaks.

ADEM is an immune complex-mediated injury to the brain usually seen during the convalescent phase of viral infection or after vaccination^(24)^. Classically, there is involvement of the cerebral white matter and deep gray matter nuclei in ADEM; although hemorrhagic foci have been reported, they are relatively uncommon. Therefore the MRI findings of ADEM and demyelinating dengue-induced hemorrhagic encephalitis might be similar. However, the temporal relationship between the occurrence of lesions and the acute febrile illness, together with positive serology for dengue, makes the diagnosis of post-dengue ADEM more plausible^(25,26)^. During the acute phase of the illness, our patients presented with involvement of the cerebral white matter with hemorrhagic foci scattered throughout the lesion.

**Figure 3:**
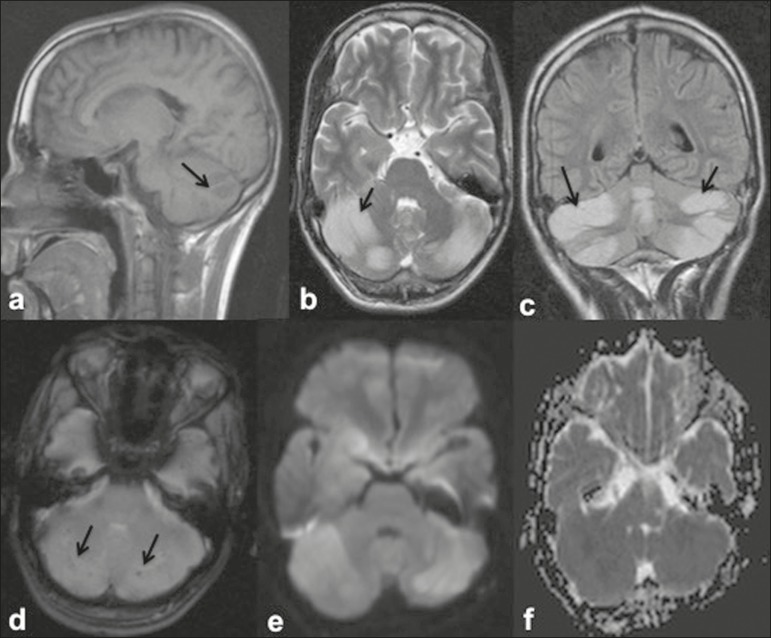
25-year-old male with dengue fever and acute cerebellitis. Sagittal T1-weighted image (a) showing subtle areas of hypointensity involving the cerebellum (arrow), which is hyperintense on an axial T2-weighted image (b) and a coronal FLAIR image (c). SWI image (d) showing subtle punctate hemorrhagic foci (arrows). DWI image at a b factor of 1000 s/mm^2^ (e) showing high signal intensity in the same region, which appears dark on an apparent diffusion coefficient map (f), suggesting restricted diffusion.

Our study included a patient with a high-grade fever and a rash who presented with acute ataxia, dysmetria, and nystagmus. In that patient, the MRI scans showed signal alteration localized to the cerebellar cortex, white matter, and vermis, all of which showed areas of restricted diffusion and hemorrhagic foci on DWI and SWI sequences, respectively (Figure 3). We identified only isolated case reports of patients with dengue fever presenting with symptoms of cerebellitis^(7)^. Cerebellar involvement in dengue patients has been thought to be part of a postinfectious immune-mediated process^(27)^. However, there have been reports of cerebellar infection in patients with dengue, in whom pathological studies have revealed the presence of viral antigen in the cerebellar cells^(28)^. Therefore, early cerebellar involvement (during the acute phase of the disease) could indicate primary cerebellar infection. Other causes of acute viral cerebellitis include infection with Epstein-Barr virus, Coxsackie virus, or varicella-zoster virus, as well as measles, mumps, herpes, and, rarely, Japanese encephalitis^(29)^ In our study, the patient with cerebellitis had thrombocytopenia, showed positive serology for dengue, and presented during the acute phase of febrile illness; hence, a diagnosis of dengue cerebellitis was most likely.

## CONCLUSION

MRI is more sensitive than computed tomography in the evaluation of dengue fever patients with early neurological manifestations. MRI can help confirm dengue encephalitis and determine the sites of involvement with high accuracy. The commonly affected areas in dengue encephalitis are the basal ganglia, thalamus, cerebellum, cerebral cortex, and white matter. Most of the lesions encountered show restricted diffusion on DWI, hemorrhagic foci on SWI, and minimal heterogenous enhancement on contrast-enhanced images. In an appropriate clinical setting, MRI can help corroborate the diagnosis of dengue encephalitis. Although there is no specific treatment for dengue infection, early identification of neurological complications by MRI can facilitate the timely institution of supportive management in affected patients. Therefore, MRI should be an integral part of the evaluation of cases of dengue fever in which there are neurological complications.
